# 5′′-Benzyl­idene-5-chloro-1′,1′′-dimethyl-4′-phenyl­dispiro­[indoline-3,2′-pyrrolidine-3′,3′′-piperidine]-2,4′′-dione

**DOI:** 10.1107/S1600536813032765

**Published:** 2013-12-07

**Authors:** I. S. Ahmed Farag, Adel S. Girgis, A. A. Ramadan, A. M. Moustafa, Edward R. T. Tiekink

**Affiliations:** a Solid State Department, Physics Division, National Research Centre, Dokki, Giza, Egypt; b Pesticide Chemistry Department, National Research Centre, Dokki, Giza 12622, Egypt; c Physics Department, Faculty of Science, Helwan University, Helwan, Cairo, Egypt; d Department of Chemistry, University of Malaya, 50603 Kuala Lumpur, Malaysia

## Abstract

The title compound, C_30_H_28_ClN_3_O_2_, features two spiro links, one connecting the piperidine and pyrrolidine rings, and the other connecting the pyrrolidine ring and indole residue. The configuration about the ethene bond is *E*. The piperidine ring adopts a half-chair conformation where the C atom connected to the spiro-C atom lies 0.713 (3) Å out of the plane of the remaining five atoms (r.m.s. deviation = 0.086 Å). The pyrrolidine ring has an envelope conformation with the flap atom being the methyl­ene C atom. Centrosymmetric eight-membered {⋯HNCO}_2_ amide synthons feature in the crystal packing. These are consolidated into a three-dimensional architecture by phen­yl–pyrrolidine C—H⋯N and chloro­benzene–pyrrolidine-bound phenyl C—H⋯π inter­actions.

## Related literature   

For the biological activity of related spiro­pyrrolidine analogues, see: Girgis *et al.* (2012 ▶); Kumar *et al.* (2008 ▶). For related structural studies, see: Moustafa *et al.* (2012 ▶). For the synthesis of the precursor mol­ecule, see: Al-Omary *et al.* (2012 ▶).
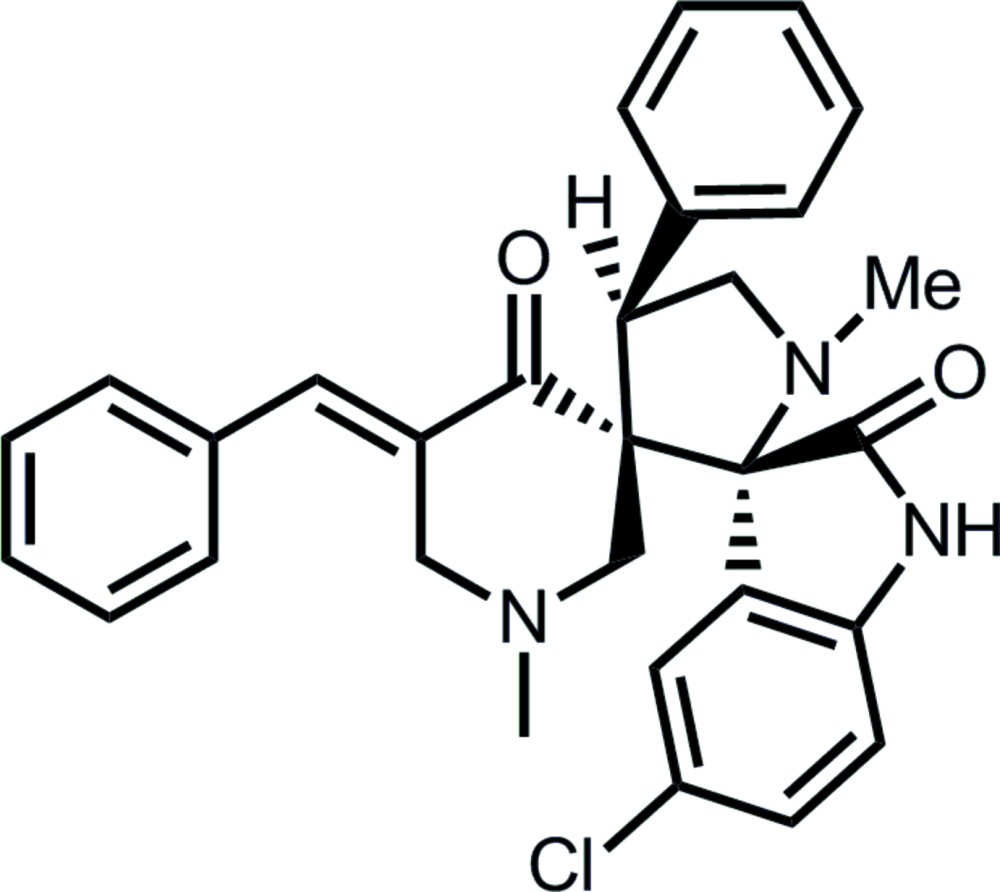


## Experimental   

### 

#### Crystal data  

C_30_H_28_ClN_3_O_2_
*M* *_r_* = 498.00
Monoclinic, 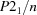

*a* = 10.5028 (3) Å
*b* = 20.4117 (6) Å
*c* = 11.9951 (4) Å
β = 94.877 (1)°
*V* = 2562.20 (14) Å^3^
*Z* = 4
Mo *K*α radiation
μ = 0.18 mm^−1^
*T* = 293 K
0.52 × 0.22 × 0.15 mm

#### Data collection  

Nonius 590 KappaCCD diffractometer
10395 measured reflections
5842 independent reflections
2547 reflections with *I* > 2σ(*I*)
*R*_int_ = 0.066

#### Refinement  

*R*[*F* ^2^ > 2σ(*F* ^2^)] = 0.052
*wR*(*F* ^2^) = 0.137
*S* = 0.94
5842 reflections
327 parameters
H-atom parameters constrained
Δρ_max_ = 0.18 e Å^−3^
Δρ_min_ = −0.35 e Å^−3^

### 

Data collection: *COLLECT* (Hooft, 1998 ▶); cell refinement: *DENZO* (Otwinowski & Minor, 1997 ▶) and *COLLECT*; data reduction: *DENZO* and *COLLECT*; program(s) used to solve structure: *SHELXS97* (Sheldrick, 2008 ▶); program(s) used to refine structure: *SHELXL97* (Sheldrick, 2008 ▶); molecular graphics: *ORTEP-3 for Windows* (Farrugia, 2012 ▶) and *DIAMOND* (Brandenburg, 2006 ▶); software used to prepare material for publication: *publCIF* (Westrip, 2010 ▶).

## Supplementary Material

Crystal structure: contains datablock(s) general, I. DOI: 10.1107/S1600536813032765/hg5367sup1.cif

Structure factors: contains datablock(s) I. DOI: 10.1107/S1600536813032765/hg5367Isup2.hkl

Additional supporting information:  crystallographic information; 3D view; checkCIF report

## Figures and Tables

**Table 1:** Hydrogen-bond geometry (Å, °) *Cg*1 is the centroid of the C25–C30 ring.

*D*—H⋯*A*	*D*—H	H⋯*A*	*D*⋯*A*	*D*—H⋯*A*
N3—H3*n*⋯O2^i^	0.86	2.01	2.854 (3)	165
C14—H14⋯N2^ii^	0.93	2.58	3.480 (4)	163
C20—H20⋯*Cg*1^iii^	0.93	2.70	3.268 (3)	121

Symmetry codes: (i) 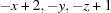; (ii) 
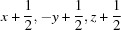
; (iii) 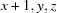.

**Fig. 1.**
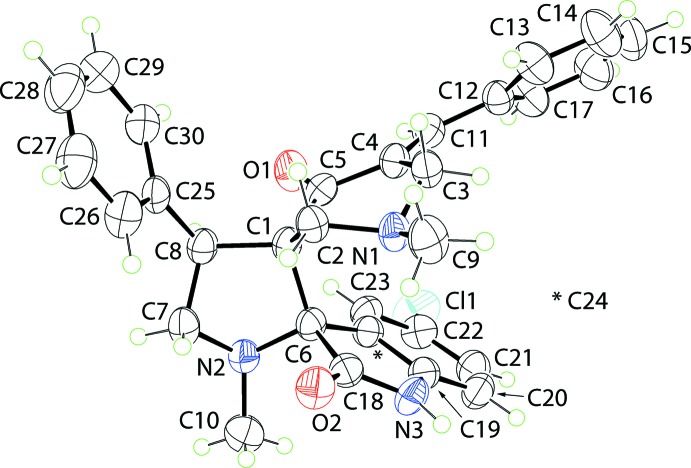
The molecular structure of (I) showing the atom-labelling scheme and displacement ellipsoids at the 50% probability level.

**Fig. 2.**
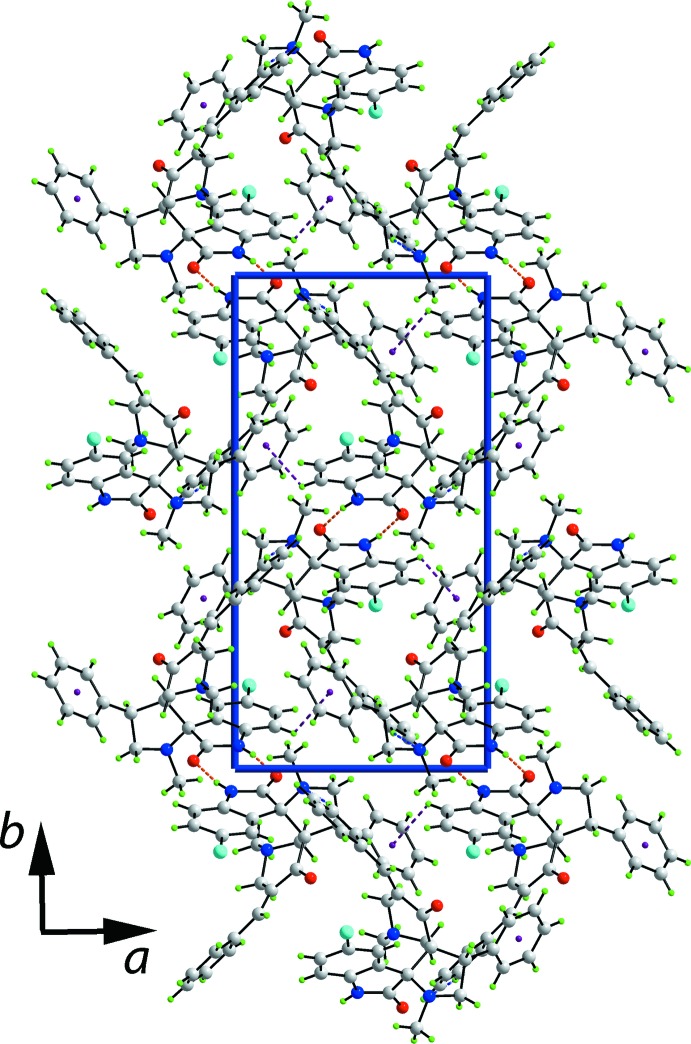
A view in projection down the c axis of the unit-cell contents for (I). The N—H···O, C—H···N and C—H···π interactions are shown as orange, blue and purple dashed lines, respectively.

## supplementary crystallographic information

### 2.1. Synthesis and crystallization

A mixture of equimolar amounts of 3*E*,5*E*-1-methyl-3,5-bis­(phenyl­methyl­idene)-4-piperidone (5 mmol), prepared by a literature procedure (Al-Omary *et al.*, 2012), 5-chloro­isatin and sarcosine in absolute ethanol (25 ml) was boiled under reflux (TLC monitoring). The separated solid was collected and crystallized from *n*-butanol affording (I). Reaction time 9 h. Colourless crystals. *M*.pt: 512–514 K. Yield 88%. Anal. Calcd. for C_30_H_28_ClN_3_O_2_ (498.03): C, 72.35; H, 5.67; N, 8.44. Found: C, 72.56; H, 5.81; N, 8.67. IR: ν_max_/cm^-1^: 3168 (N—H); 1688 (C═O); 1597, 1457 (C═C).

### 2.2. Refinement

The C-bound H atoms were geometrically placed (C—H = 0.93–0.98 Å) and refined as riding with *U_iso_*(H) = 1.2–1.5*U_eq_*(C). The N-bound H-atoms were treated similarly with N—H = 0.86 Å, and with *U_iso_*(H) = 1.2*U_eq_*(N).

### 3. Results and discussion

In connection with on-going studies of spiro­pyrrolidine derivatives (Girgis *et al.* 2012; Moustafa *et al.* 2012), the title compound, (I), was synthesised and characterised crystallographically. These compounds have biological activity and the structure of the skeltal structure is well established (Kumar *et al.* 2008).

There are two spiro links in the molecule, Fig. 1, i) where the piperidine and pyrrolidine rings are connected at C1, and ii) where the pyrrolidine ring and indole residue are connected at C6. The phenyl­methyl­idene functional group is connected to the piperidine ring at position C4 while the pyrrolidine-bound aryl ring is attached at C8. The conformation about the C4═C11 double bond is *E*. The sum of the angles around the piperidine-N1 atom is approximately 333° confirming its *sp*^3^ character. The piperidine ring adopts a half-chair conformation where the C2 atom lies 0.713 (3) Å out of the plane of the remaining five atoms (r.m.s. deviation = 0.086 Å). The C6 and C8 atoms occupy axial and equatorial positions with respect to the piperidine ring, the phenyl­methyl­idene residue occupies an equatorial position, and the N-bound methyl substituent is equatorial. The pyrrolidine ring has an envelope conformation with the flap atom being the C7 atom which lies 0.648 (3) Å out of the plane of the remaining four atoms (r.m.s. deviation = 0.026 Å). Finally, the indole fused ring system is planar with a r.m.s. deviation = 0.051 Å.

The most prominent feature of the crystal packing of is the formation of centrosymmetric eight-membered {···HNCO}_2_ synthons owing to the self-association of molecules *via* hydrogen bonding between amide groups, Table 1. The dimers are connected into a supra­molecular chain parallel to the *b* axis by phenyl-C–H···N (pyrrolidine) inter­actions and these are consolidated into a three-dimensional architecture by (chloro­benzene)C—H···π (pyrrolidine-bound phenyl), edge-to-face, inter­actions; a view of the unit cell contents is shown in Fig. 2.

### Crystal data

**Table:**

C_30_H_28_ClN_3_O_2_	*F*(000) = 1048
*M**_r_* = 498.00	*D*_x_ = 1.291 Mg m^−^^3^
Monoclinic, *P*2_1_/*n*	Mo *K*α radiation, λ = 0.71073 Å
Hall symbol: -P 2yn	Cell parameters from 5029 reflections
*a* = 10.5028 (3) Å	θ = 2.9–27.5°
*b* = 20.4117 (6) Å	µ = 0.18 mm^−^^1^
*c* = 11.9951 (4) Å	*T* = 293 K
β = 94.877 (1)°	Block, colourless
*V* = 2562.20 (14) Å^3^	0.52 × 0.22 × 0.15 mm
*Z* = 4	

### Data collection

**Table:**

Nonius 590 KappaCCD diffractometer	2547 reflections with *I* > 2σ(*I*)
Radiation source: fine-focus sealed tube	*R*_int_ = 0.066
Graphite monochromator	θ_max_ = 27.5°, θ_min_ = 3.2°
φ and ω scans	*h* = −13→13
10395 measured reflections	*k* = −24→26
5842 independent reflections	*l* = −15→15

### Refinement

**Table:**

Refinement on *F*^2^	Primary atom site location: structure-invariant direct methods
Least-squares matrix: full	Secondary atom site location: difference Fourier map
*R*[*F*^2^ > 2σ(*F*^2^)] = 0.052	Hydrogen site location: inferred from neighbouring sites
*wR*(*F*^2^) = 0.137	H-atom parameters constrained
*S* = 0.94	*w* = 1/[σ^2^(*F*_o_^2^) + (0.0569*P*)^2^] where *P* = (*F*_o_^2^ + 2*F*_c_^2^)/3
5842 reflections	(Δ/σ)_max_ = 0.001
327 parameters	Δρ_max_ = 0.18 e Å^−^^3^
0 restraints	Δρ_min_ = −0.35 e Å^−^^3^

### Special details

**Table:**

Geometry. All s.u.'s (except the s.u. in the dihedral angle between two l.s. planes) are estimated using the full covariance matrix. The cell s.u.'s are taken into account individually in the estimation of s.u.'s in distances, angles and torsion angles; correlations between s.u.'s in cell parameters are only used when they are defined by crystal symmetry. An approximate (isotropic) treatment of cell s.u.'s is used for estimating s.u.'s involving l.s. planes.
Refinement. Refinement of *F*^2^ against ALL reflections. The weighted *R*-factor *wR* and goodness of fit *S* are based on *F*^2^, conventional *R*-factors *R* are based on *F*, with *F* set to zero for negative *F*^2^. The threshold expression of *F*^2^ > 2σ(*F*^2^) is used only for calculating *R*-factors(gt) *etc*. and is not relevant to the choice of reflections for refinement. *R*-factors based on *F*^2^ are statistically about twice as large as those based on *F*, and *R*- factors based on ALL data will be even larger.

### Fractional atomic coordinates and isotropic or equivalent isotropic displacement parameters (Å^2^)

**Table:**

	*x*	*y*	*z*	*U*_iso_*/*U*_eq_	
Cl1	1.05716 (7)	0.16826 (4)	−0.06845 (7)	0.0777 (3)	
O1	0.70008 (15)	0.22207 (8)	0.17686 (15)	0.0518 (5)	
O2	0.83631 (15)	0.01572 (8)	0.45243 (14)	0.0501 (5)	
N1	0.87248 (16)	0.16478 (9)	0.47693 (16)	0.0395 (5)	
N2	0.73065 (16)	0.03868 (9)	0.21124 (16)	0.0425 (5)	
N3	1.01454 (17)	0.04625 (9)	0.36752 (17)	0.0441 (5)	
H3n	1.0711	0.0286	0.4142	0.053*	
C1	0.72419 (19)	0.13839 (11)	0.31799 (19)	0.0354 (6)	
C2	0.7398 (2)	0.14837 (11)	0.44474 (19)	0.0389 (6)	
H2A	0.6845	0.1835	0.4659	0.047*	
H2B	0.7165	0.1086	0.4825	0.047*	
C3	0.8953 (2)	0.23259 (11)	0.4469 (2)	0.0425 (6)	
H3A	0.9855	0.2423	0.4618	0.051*	
H3B	0.8485	0.2612	0.4935	0.051*	
C4	0.8552 (2)	0.24660 (11)	0.32605 (19)	0.0370 (6)	
C5	0.7557 (2)	0.20414 (11)	0.2654 (2)	0.0377 (6)	
C6	0.81725 (19)	0.08258 (11)	0.27869 (19)	0.0364 (6)	
C7	0.6081 (2)	0.04033 (11)	0.2593 (2)	0.0463 (7)	
H7A	0.6121	0.0177	0.3307	0.056*	
H7B	0.5409	0.0212	0.2091	0.056*	
C8	0.58871 (19)	0.11328 (11)	0.2732 (2)	0.0391 (6)	
H8	0.5710	0.1315	0.1979	0.047*	
C9	0.9106 (2)	0.15392 (13)	0.5956 (2)	0.0562 (7)	
H9A	0.8554	0.1784	0.6401	0.084*	
H9B	0.9972	0.1681	0.6124	0.084*	
H9C	0.9041	0.1081	0.6124	0.084*	
C10	0.7805 (2)	−0.02622 (13)	0.1881 (2)	0.0615 (8)	
H10A	0.8615	−0.0219	0.1573	0.092*	
H10B	0.7216	−0.0485	0.1354	0.092*	
H10C	0.7911	−0.0510	0.2562	0.092*	
C11	0.9050 (2)	0.29475 (11)	0.2683 (2)	0.0423 (6)	
H11	0.8740	0.2975	0.1935	0.051*	
C12	1.0017 (2)	0.34419 (11)	0.3053 (2)	0.0408 (6)	
C13	1.0215 (2)	0.36746 (13)	0.4136 (2)	0.0546 (7)	
H13	0.9709	0.3519	0.4678	0.066*	
C14	1.1149 (3)	0.41337 (14)	0.4432 (3)	0.0657 (8)	
H14	1.1273	0.4279	0.5168	0.079*	
C15	1.1892 (3)	0.43743 (14)	0.3646 (3)	0.0658 (8)	
H15	1.2537	0.4674	0.3849	0.079*	
C16	1.1685 (3)	0.41725 (14)	0.2555 (3)	0.0667 (9)	
H16	1.2169	0.4347	0.2012	0.080*	
C17	1.0760 (2)	0.37125 (12)	0.2266 (2)	0.0540 (7)	
H17	1.0627	0.3579	0.1524	0.065*	
C18	0.8882 (2)	0.04577 (11)	0.3795 (2)	0.0407 (6)	
C19	1.0428 (2)	0.07885 (11)	0.2700 (2)	0.0385 (6)	
C20	1.1587 (2)	0.08588 (12)	0.2266 (2)	0.0485 (7)	
H20	1.2336	0.0717	0.2662	0.058*	
C21	1.1613 (2)	0.11466 (12)	0.1225 (2)	0.0524 (7)	
H21	1.2388	0.1201	0.0915	0.063*	
C22	1.0499 (2)	0.13532 (12)	0.0645 (2)	0.0478 (7)	
C23	0.9324 (2)	0.12955 (11)	0.1090 (2)	0.0435 (6)	
H23	0.8577	0.1440	0.0693	0.052*	
C24	0.9298 (2)	0.10180 (11)	0.2136 (2)	0.0368 (6)	
C25	0.4792 (2)	0.13400 (12)	0.3399 (2)	0.0394 (6)	
C26	0.4383 (2)	0.09737 (14)	0.4274 (2)	0.0544 (7)	
H26	0.4793	0.0582	0.4477	0.065*	
C27	0.3363 (2)	0.11858 (17)	0.4853 (2)	0.0652 (8)	
H27	0.3094	0.0935	0.5436	0.078*	
C28	0.2756 (3)	0.17634 (18)	0.4566 (3)	0.0722 (9)	
H28	0.2083	0.1907	0.4959	0.087*	
C29	0.3145 (3)	0.21279 (15)	0.3698 (3)	0.0701 (9)	
H29	0.2731	0.2518	0.3497	0.084*	
C30	0.4153 (2)	0.19161 (13)	0.3119 (2)	0.0553 (7)	
H30	0.4406	0.2167	0.2529	0.066*	

### Atomic displacement parameters (Å^2^)

**Table:**

	*U*^11^	*U*^22^	*U*^33^	*U*^12^	*U*^13^	*U*^23^
Cl1	0.0823 (5)	0.0892 (6)	0.0655 (6)	0.0142 (4)	0.0285 (4)	0.0289 (4)
O1	0.0515 (10)	0.0547 (11)	0.0467 (12)	−0.0077 (8)	−0.0102 (9)	0.0147 (9)
O2	0.0486 (10)	0.0543 (11)	0.0466 (12)	−0.0018 (9)	−0.0003 (9)	0.0159 (9)
N1	0.0364 (11)	0.0465 (13)	0.0341 (13)	−0.0047 (9)	−0.0047 (9)	0.0055 (10)
N2	0.0362 (11)	0.0430 (13)	0.0475 (14)	−0.0005 (10)	−0.0004 (10)	−0.0057 (10)
N3	0.0350 (11)	0.0538 (14)	0.0420 (14)	0.0067 (10)	−0.0051 (10)	0.0087 (10)
C1	0.0314 (12)	0.0416 (15)	0.0326 (15)	−0.0011 (11)	−0.0019 (10)	0.0045 (12)
C2	0.0385 (13)	0.0408 (15)	0.0369 (16)	−0.0052 (11)	0.0010 (11)	0.0029 (12)
C3	0.0380 (13)	0.0440 (16)	0.0445 (17)	−0.0070 (11)	−0.0019 (11)	0.0012 (13)
C4	0.0345 (13)	0.0433 (15)	0.0328 (15)	−0.0015 (11)	0.0004 (11)	0.0029 (12)
C5	0.0338 (13)	0.0426 (15)	0.0365 (17)	0.0044 (11)	0.0027 (12)	0.0039 (13)
C6	0.0344 (12)	0.0389 (14)	0.0349 (15)	−0.0007 (11)	−0.0032 (11)	0.0030 (11)
C7	0.0381 (14)	0.0516 (17)	0.0479 (17)	−0.0072 (12)	−0.0034 (12)	−0.0004 (13)
C8	0.0333 (12)	0.0450 (16)	0.0378 (15)	−0.0002 (11)	−0.0033 (11)	0.0037 (12)
C9	0.0597 (16)	0.0632 (19)	0.0434 (19)	−0.0088 (14)	−0.0096 (13)	0.0069 (14)
C10	0.0593 (17)	0.0547 (19)	0.070 (2)	−0.0006 (14)	0.0051 (15)	−0.0124 (15)
C11	0.0415 (14)	0.0472 (16)	0.0379 (16)	0.0002 (13)	0.0014 (12)	−0.0011 (13)
C12	0.0403 (13)	0.0411 (15)	0.0405 (17)	−0.0034 (12)	0.0010 (12)	0.0031 (13)
C13	0.0698 (18)	0.0511 (17)	0.0429 (19)	−0.0159 (15)	0.0052 (14)	0.0042 (14)
C14	0.085 (2)	0.060 (2)	0.049 (2)	−0.0203 (17)	−0.0112 (16)	0.0024 (16)
C15	0.0552 (18)	0.060 (2)	0.080 (3)	−0.0204 (14)	−0.0069 (17)	0.0028 (18)
C16	0.0612 (18)	0.066 (2)	0.076 (3)	−0.0189 (16)	0.0223 (17)	−0.0040 (18)
C17	0.0590 (16)	0.0555 (17)	0.0490 (19)	−0.0108 (15)	0.0136 (14)	−0.0047 (15)
C18	0.0404 (15)	0.0385 (15)	0.0424 (17)	0.0005 (12)	−0.0014 (12)	0.0041 (13)
C19	0.0393 (14)	0.0391 (15)	0.0363 (16)	0.0001 (11)	−0.0004 (12)	−0.0020 (12)
C20	0.0363 (14)	0.0548 (17)	0.0537 (19)	0.0049 (12)	0.0003 (13)	−0.0009 (14)
C21	0.0422 (15)	0.0568 (18)	0.060 (2)	−0.0043 (13)	0.0140 (14)	−0.0018 (15)
C22	0.0522 (16)	0.0494 (17)	0.0430 (18)	0.0022 (13)	0.0120 (14)	0.0046 (13)
C23	0.0407 (14)	0.0439 (16)	0.0450 (17)	0.0038 (12)	−0.0009 (12)	0.0048 (13)
C24	0.0375 (13)	0.0371 (14)	0.0354 (16)	0.0005 (11)	0.0001 (11)	−0.0005 (12)
C25	0.0291 (12)	0.0450 (16)	0.0430 (17)	−0.0059 (12)	−0.0040 (11)	−0.0003 (13)
C26	0.0436 (15)	0.0665 (19)	0.0527 (19)	−0.0041 (14)	0.0013 (14)	0.0088 (15)
C27	0.0514 (17)	0.095 (3)	0.050 (2)	−0.0111 (17)	0.0109 (15)	−0.0051 (17)
C28	0.0448 (17)	0.096 (3)	0.077 (3)	−0.0018 (18)	0.0135 (16)	−0.030 (2)
C29	0.0516 (18)	0.063 (2)	0.097 (3)	0.0026 (15)	0.0102 (18)	−0.0110 (19)
C30	0.0409 (15)	0.0537 (18)	0.071 (2)	−0.0044 (14)	0.0042 (14)	−0.0004 (15)

### Geometric parameters (Å, º)

**Table:**

Cl1—C22	1.738 (3)	C10—H10C	0.9600
O1—C5	1.224 (3)	C11—C12	1.473 (3)
O2—C18	1.232 (3)	C11—H11	0.9300
N1—C2	1.453 (3)	C12—C13	1.382 (3)
N1—C3	1.455 (3)	C12—C17	1.390 (3)
N1—C9	1.463 (3)	C13—C14	1.381 (4)
N2—C7	1.454 (3)	C13—H13	0.9300
N2—C10	1.460 (3)	C14—C15	1.366 (4)
N2—C6	1.470 (3)	C14—H14	0.9300
N3—C18	1.347 (3)	C15—C16	1.371 (4)
N3—C19	1.400 (3)	C15—H15	0.9300
N3—H3n	0.8600	C16—C17	1.374 (3)
C1—C5	1.531 (3)	C16—H16	0.9300
C1—C2	1.529 (3)	C17—H17	0.9300
C1—C8	1.564 (3)	C19—C20	1.371 (3)
C1—C6	1.598 (3)	C19—C24	1.396 (3)
C2—H2A	0.9700	C20—C21	1.382 (3)
C2—H2B	0.9700	C20—H20	0.9300
C3—C4	1.502 (3)	C21—C22	1.375 (3)
C3—H3A	0.9700	C21—H21	0.9300
C3—H3B	0.9700	C22—C23	1.391 (3)
C4—C11	1.334 (3)	C23—C24	1.380 (3)
C4—C5	1.498 (3)	C23—H23	0.9300
C6—C24	1.522 (3)	C25—C30	1.381 (3)
C6—C18	1.559 (3)	C25—C26	1.386 (3)
C7—C8	1.514 (3)	C26—C27	1.394 (3)
C7—H7A	0.9700	C26—H26	0.9300
C7—H7B	0.9700	C27—C28	1.370 (4)
C8—C25	1.516 (3)	C27—H27	0.9300
C8—H8	0.9800	C28—C29	1.370 (4)
C9—H9A	0.9600	C28—H28	0.9300
C9—H9B	0.9600	C29—C30	1.384 (4)
C9—H9C	0.9600	C29—H29	0.9300
C10—H10A	0.9600	C30—H30	0.9300
C10—H10B	0.9600		
			
C2—N1—C3	109.14 (18)	H10B—C10—H10C	109.5
C2—N1—C9	113.61 (17)	C4—C11—C12	129.7 (2)
C3—N1—C9	110.35 (18)	C4—C11—H11	115.2
C7—N2—C10	116.12 (18)	C12—C11—H11	115.2
C7—N2—C6	107.14 (17)	C13—C12—C17	117.0 (2)
C10—N2—C6	116.30 (18)	C13—C12—C11	124.3 (2)
C18—N3—C19	111.96 (19)	C17—C12—C11	118.7 (2)
C18—N3—H3n	124.0	C14—C13—C12	121.5 (2)
C19—N3—H3n	124.0	C14—C13—H13	119.3
C5—C1—C2	106.50 (19)	C12—C13—H13	119.3
C5—C1—C8	111.60 (18)	C15—C14—C13	120.1 (3)
C2—C1—C8	113.73 (17)	C15—C14—H14	120.0
C5—C1—C6	110.15 (16)	C13—C14—H14	120.0
C2—C1—C6	111.83 (18)	C14—C15—C16	119.8 (3)
C8—C1—C6	103.10 (17)	C14—C15—H15	120.1
N1—C2—C1	108.31 (17)	C16—C15—H15	120.1
N1—C2—H2A	110.0	C15—C16—C17	119.9 (3)
C1—C2—H2A	110.0	C15—C16—H16	120.1
N1—C2—H2B	110.0	C17—C16—H16	120.1
C1—C2—H2B	110.0	C16—C17—C12	121.7 (3)
H2A—C2—H2B	108.4	C16—C17—H17	119.2
N1—C3—C4	112.41 (19)	C12—C17—H17	119.2
N1—C3—H3A	109.1	O2—C18—N3	125.4 (2)
C4—C3—H3A	109.1	O2—C18—C6	125.4 (2)
N1—C3—H3B	109.1	N3—C18—C6	108.9 (2)
C4—C3—H3B	109.1	C20—C19—C24	121.8 (2)
H3A—C3—H3B	107.9	C20—C19—N3	128.7 (2)
C11—C4—C5	117.5 (2)	C24—C19—N3	109.40 (19)
C11—C4—C3	123.4 (2)	C19—C20—C21	118.2 (2)
C5—C4—C3	119.0 (2)	C19—C20—H20	120.9
O1—C5—C4	120.7 (2)	C21—C20—H20	120.9
O1—C5—C1	121.0 (2)	C22—C21—C20	120.4 (2)
C4—C5—C1	118.3 (2)	C22—C21—H21	119.8
N2—C6—C24	110.25 (18)	C20—C21—H21	119.8
N2—C6—C18	111.47 (18)	C21—C22—C23	121.6 (2)
C24—C6—C18	100.44 (17)	C21—C22—Cl1	118.77 (19)
N2—C6—C1	103.40 (16)	C23—C22—Cl1	119.6 (2)
C24—C6—C1	119.26 (18)	C24—C23—C22	118.1 (2)
C18—C6—C1	112.25 (18)	C24—C23—H23	121.0
N2—C7—C8	101.46 (17)	C22—C23—H23	121.0
N2—C7—H7A	111.5	C23—C24—C19	119.8 (2)
C8—C7—H7A	111.5	C23—C24—C6	130.4 (2)
N2—C7—H7B	111.5	C19—C24—C6	109.3 (2)
C8—C7—H7B	111.5	C30—C25—C26	117.9 (2)
H7A—C7—H7B	109.3	C30—C25—C8	118.9 (2)
C7—C8—C25	116.62 (19)	C26—C25—C8	123.2 (2)
C7—C8—C1	103.47 (17)	C25—C26—C27	120.7 (3)
C25—C8—C1	115.89 (19)	C25—C26—H26	119.7
C7—C8—H8	106.7	C27—C26—H26	119.7
C25—C8—H8	106.7	C28—C27—C26	120.2 (3)
C1—C8—H8	106.7	C28—C27—H27	119.9
N1—C9—H9A	109.5	C26—C27—H27	119.9
N1—C9—H9B	109.5	C29—C28—C27	119.7 (3)
H9A—C9—H9B	109.5	C29—C28—H28	120.1
N1—C9—H9C	109.5	C27—C28—H28	120.1
H9A—C9—H9C	109.5	C28—C29—C30	120.1 (3)
H9B—C9—H9C	109.5	C28—C29—H29	119.9
N2—C10—H10A	109.5	C30—C29—H29	119.9
N2—C10—H10B	109.5	C25—C30—C29	121.4 (3)
H10A—C10—H10B	109.5	C25—C30—H30	119.3
N2—C10—H10C	109.5	C29—C30—H30	119.3
H10A—C10—H10C	109.5		
			
C3—N1—C2—C1	76.1 (2)	C11—C12—C13—C14	179.2 (2)
C9—N1—C2—C1	−160.35 (19)	C12—C13—C14—C15	0.8 (4)
C5—C1—C2—N1	−62.7 (2)	C13—C14—C15—C16	1.8 (4)
C8—C1—C2—N1	173.98 (18)	C14—C15—C16—C17	−2.2 (4)
C6—C1—C2—N1	57.7 (2)	C15—C16—C17—C12	0.0 (4)
C2—N1—C3—C4	−53.4 (2)	C13—C12—C17—C16	2.4 (4)
C9—N1—C3—C4	−178.89 (18)	C11—C12—C17—C16	−179.5 (2)
N1—C3—C4—C11	−155.0 (2)	C19—N3—C18—O2	173.4 (2)
N1—C3—C4—C5	24.1 (3)	C19—N3—C18—C6	−0.1 (3)
C11—C4—C5—O1	−17.4 (3)	N2—C6—C18—O2	−56.8 (3)
C3—C4—C5—O1	163.4 (2)	C24—C6—C18—O2	−173.6 (2)
C11—C4—C5—C1	163.4 (2)	C1—C6—C18—O2	58.7 (3)
C3—C4—C5—C1	−15.7 (3)	N2—C6—C18—N3	116.7 (2)
C2—C1—C5—O1	−145.8 (2)	C24—C6—C18—N3	0.0 (2)
C8—C1—C5—O1	−21.2 (3)	C1—C6—C18—N3	−127.80 (19)
C6—C1—C5—O1	92.7 (2)	C18—N3—C19—C20	−175.8 (2)
C2—C1—C5—C4	33.3 (2)	C18—N3—C19—C24	0.3 (3)
C8—C1—C5—C4	157.97 (19)	C24—C19—C20—C21	−2.2 (4)
C6—C1—C5—C4	−88.1 (2)	N3—C19—C20—C21	173.5 (2)
C7—N2—C6—C24	−160.87 (19)	C19—C20—C21—C22	−0.2 (4)
C10—N2—C6—C24	67.3 (3)	C20—C21—C22—C23	1.6 (4)
C7—N2—C6—C18	88.5 (2)	C20—C21—C22—Cl1	−177.5 (2)
C10—N2—C6—C18	−43.3 (3)	C21—C22—C23—C24	−0.5 (4)
C7—N2—C6—C1	−32.3 (2)	Cl1—C22—C23—C24	178.52 (19)
C10—N2—C6—C1	−164.09 (19)	C22—C23—C24—C19	−1.8 (3)
C5—C1—C6—N2	−114.14 (19)	C22—C23—C24—C6	−173.0 (2)
C2—C1—C6—N2	127.64 (18)	C20—C19—C24—C23	3.2 (3)
C8—C1—C6—N2	5.1 (2)	N3—C19—C24—C23	−173.2 (2)
C5—C1—C6—C24	8.6 (3)	C20—C19—C24—C6	176.1 (2)
C2—C1—C6—C24	−109.6 (2)	N3—C19—C24—C6	−0.3 (3)
C8—C1—C6—C24	127.8 (2)	N2—C6—C24—C23	54.4 (3)
C5—C1—C6—C18	125.6 (2)	C18—C6—C24—C23	172.1 (2)
C2—C1—C6—C18	7.4 (2)	C1—C6—C24—C23	−64.9 (3)
C8—C1—C6—C18	−115.19 (19)	N2—C6—C24—C19	−117.5 (2)
C10—N2—C7—C8	179.1 (2)	C18—C6—C24—C19	0.2 (2)
C6—N2—C7—C8	47.2 (2)	C1—C6—C24—C19	123.2 (2)
N2—C7—C8—C25	−170.10 (19)	C7—C8—C25—C30	−147.6 (2)
N2—C7—C8—C1	−41.6 (2)	C1—C8—C25—C30	90.2 (3)
C5—C1—C8—C7	140.24 (19)	C7—C8—C25—C26	31.5 (3)
C2—C1—C8—C7	−99.2 (2)	C1—C8—C25—C26	−90.7 (3)
C6—C1—C8—C7	22.0 (2)	C30—C25—C26—C27	−0.4 (4)
C5—C1—C8—C25	−90.8 (2)	C8—C25—C26—C27	−179.5 (2)
C2—C1—C8—C25	29.7 (3)	C25—C26—C27—C28	−0.4 (4)
C6—C1—C8—C25	150.97 (19)	C26—C27—C28—C29	0.8 (4)
C5—C4—C11—C12	179.0 (2)	C27—C28—C29—C30	−0.5 (4)
C3—C4—C11—C12	−1.9 (4)	C26—C25—C30—C29	0.7 (4)
C4—C11—C12—C13	−28.2 (4)	C8—C25—C30—C29	179.9 (2)
C4—C11—C12—C17	153.9 (2)	C28—C29—C30—C25	−0.3 (4)
C17—C12—C13—C14	−2.9 (4)		

### Hydrogen-bond geometry (Å, º)

Cg1 is the centroid of the C25–C30 ring.

**Table:**

*D*—H···*A*	*D*—H	H···*A*	*D*···*A*	*D*—H···*A*
N3—H3*n*···O2^i^	0.86	2.01	2.854 (3)	165
C14—H14···N2^ii^	0.93	2.58	3.480 (4)	163
C20—H20···*Cg*1^iii^	0.93	2.70	3.268 (3)	121

Symmetry codes: (i) −*x*+2, −*y*, −*z*+1; (ii) *x*+1/2, −*y*+1/2, *z*+1/2; (iii) *x*+1, *y*, *z*.

## References

[bb1] Al-Omary, F. A. M., Hassan, G. S., El-Messery, S. M. & El-Subbagh, H. I. (2012). *Eur. J. Med. Chem.* **47**, 65–72. 10.1016/j.ejmech.2011.10.023 22056277

[bb2] Brandenburg, K. (2006). *DIAMOND* Crystal Impact GbR, Bonn, Germany.

[bb3] Farrugia, L. J. (2012). *J. Appl. Cryst.* **45**, 849–854.

[bb4] Girgis, A. S., Tala, S. R., Oliferenko, P. V., Oliferenko, A. A. & Katritzky, A. R. (2012). *Eur. J. Med. Chem.* **50** 1–8. 10.1016/j.ejmech.2011.11.034 22365409

[bb5] Hooft, R. W. W. (1998). *COLLECT* Nonius BV, Delft, The Netherlands.

[bb6] Kumar, R. R., Perumal, S., Senthilkumar, P., Yoeeswair, P. & Sriram, D. (2008). *J. Med. Chem.* **51**, 5731–5735. 10.1021/jm800545k 18714980

[bb7] Moustafa, A. M., Girgis, A. S., Shalaby, S. M. & Tiekink, E. R. T. (2012). *Acta Cryst.* E**68**, o2197–o2198. 10.1107/S1600536812028012 PMC3393996 22798861

[bb8] Otwinowski, Z. & Minor, W. (1997). *Methods in Enzymology*, Vol. 276, *Macromolecular Crystallography*, Part A, edited by C. W. Carter Jr & R. M. Sweet, pp. 307–326. New York: Academic Press.

[bb9] Sheldrick, G. M. (2008). *Acta Cryst.* A**64**, 112–122. 10.1107/S0108767307043930 18156677

[bb10] Westrip, S. P. (2010). *J. Appl. Cryst.* **43**, 920–925.

